# Correction: Isolation, Purification and Properties of an R-Phycocyanin from the Phycobilisomes of a Marine Red Macroalga *Polysiphonia urceolata*

**DOI:** 10.1371/journal.pone.0101724

**Published:** 2014-07-03

**Authors:** The PLOS ONE Staff

## Notice of Republication

This article was republished on May 16, 2014, to include missing formulas. Please download this article again to view the correct version. The originally published, uncorrected article and the republished, corrected article are provided here for reference.

## Supporting Information

File S1 Originally published, uncorrected article.
(PDF)

File S2 Republished corrected article.
(PDF)
