# Supplementary material for: Correction: Isolation, Purification and Properties of an R-Phycocyanin from the Phycobilisomes of a Marine Red Macroalga Polysiphonia urceolata
Source: PLoS One. 2014 Jul 3;9(7):e101724. doi: 10.1371/journal.pone.0101724 (PMC4081778; doi:10.1371/journal.pone.0101724)
Supplement: File S2 — Republished corrected article. (PDF) [file pone.0101724.s002.pdf]

Isolation, Purification and Properties of an R-Phycocyanin from the Phycobilisomes of a Marine Red Macroalga *Polysiphonia urceolata*

Lu Wang[1], Yanyan Qu[1], Xuejun Fu[1], Mingri Zhao[1], Shumei Wang[2], Li Sun[1]*

1 College of Life Sciences, Yantai University, Yantai, Shandong, People's Republic of China, **2** College of Photo-Electronic Information Science and Technology, Yantai University, Yantai, People's Republic of China

Abstract

Phycobilisomes were prepared from a marine red macroalga *Polysiphonia urceolata* (*P. urceolata*) by sucrose step-gradient ultracentrifugation. From the prepared phycobilisomes, an R-phycocyanin was isolated by gel filtration on Sephadex G-150 and then purified by ion exchange chromatography on DEAE-Sepharose Fast Flow and native polyacrylamide gel electrophoresis (PAGE) performed in neutral buffer systems. The purified R-phycocyanins showed not only a homogeneous trimer of 136 kDa in gel filtration and a single band in native PAGE, but also exhibited one band at about pH 5.7 in native isoelectric focusing (IEF). By a gradient SDS-PAGE the purified R-phycocyanin was determined to contain one α subunit of 17.5 kDa ($\alpha^{17.5}$) and two β subunits of 21.3 kDa and 22.6 kDa ($\beta^{21.3}$ and $\beta^{22.6}$). The analysis from denaturing isoelectric focusing and two-dimension PAGE demonstrated that $\alpha^{17.5}$, $\beta^{21.3}$ and $\beta^{22.6}$ had their pIs of 6.4, 5.3 and 5.4, respectively. Furthermore, mass spectroscopy analysis of $\beta^{21.3}$ and $\beta^{22.6}$ by MALDI-TOF mass spectrometry demonstrated the two β subunits had differences in peptide mass fingerprinting. These results revealed that the prepared R-phycocyanins were composed of one α and two β subunits. ($\alpha_3^{17.5}\beta_2^{21.3}\beta_1^{22.6}$) and ($\alpha_3^{17.5}\beta_1^{21.3}\beta_2^{22.6}$), which have a structural foundation to show their pIs too close for them to be definitely resolved by native IEF, are postulated to be the most possible trimeric forms of the R-phycocyanins prepared from the phycobilisomes of *P. urceolata*.

Citation: Wang L, Qu Y, Fu X, Zhao M, Wang S, et al. (2014) Isolation, Purification and Properties of an R-Phycocyanin from the Phycobilisomes of a Marine Red Macroalga *Polysiphonia urceolata*. PLoS ONE 9(2): e87833. doi:10.1371/journal.pone.0087833

Editor: Elena A. Rozhkova, Argonne National Laboratory, United States of America

Received September 26, 2013; **Accepted** December 31, 2013; **Published** February 4, 2014

Copyright: © 2014 Wang et al. This is an open-access article distributed under the terms of the Creative Commons Attribution License, which permits unrestricted use, distribution, and reproduction in any medium, provided the original author and source are credited.

Funding: This work was supported by National Natural Science Foundation of China (30571720, 40976083). The funders had no role in study design, data collection and analysis, decision to publish, or preparation of the manuscript.

Competing Interests: The authors have declared that no competing interests exist.

* E-mail: sunliytu@aliyun.com

Introduction

Phycobilisomes (PBSs) exist only in prokaryotic cyanobacteria and eukaryotic red algae. Cyanobacteria are primitive oxygenic photosynthetic organisms, and the chloroplasts of eukaryotic red algae are primary plastids that evolve by means of primary endosymbiosis from a cyanobacterium after it is engulfed by a heterotrophic and eukaryotic host cell. Phycobilisomes attach on the external surface of thylakoids, where photosynthesis photoreaction takes place, and function as the main photosynthetic accessories for sun light harvesting of the two organisms [1–6]. As a supramolecular complex, PBSs commonly have two subdomains: peripheral rods and central cores. Both of the subdomains are made up of phycobiliproteins and their corresponding linker polypeptides [7–10]. The phycobiliproteins are divided into three types according to their light absorption properties: phycoerythrins (PE; $\lambda_{max} = 565$ nm), phycocyanins (PC; $\lambda_{max} = 620$ nm) and allophycocyanins (AP; $\lambda_{max} = 650$ nm) [10]. Because of their excellent spectroscopic properties, such as high absorption coefficient and strong fluorescent emission, phycobiliproteins are widely used as fluorescent probes for immunodiagnostic protein and cell labeling in biological and medical tests [11–13].

Within phycobilisomes, phycoerythrins are located at the tip of PBS rods, phycocyanins which are always adjacent to PBS cores are situated at the other end of rods, and allophycocyanins are the only type of phycobiliproteins which assemble with the aid of core linker polypeptides to form PBS cores [7–10]. When sun light is harvested by phycobilisomes, the light energy is transferred from the highest-energy chromophores of PEs via the intermediate-energy chromophores of PCs to the lowest-energy chromophores of APs with an overall quantum efficiency closed to 100%, and it is finally transferred to the chlorophyll a of photosystems [5,7–10]. Therefore, phycocyanins play an important role in mediating light energy transfer from phycoerythrins to allophycocyanins as well as in combining rod domains with core domains in PBS assembly [7–10].

Phycocyanins are oligomeric proteins and composed of two different kind subunits, α and β, which combine with each other to form monomers (αβ) [2,7–10,13,14]. Three monomers connect side by side to form a trimer, $(\alpha\beta)_3$, and two trimers may further assemble face to face to build a hexamer, $(\alpha\beta)_3{\cdot}(\alpha\beta)_3$ or $(\alpha\beta)_6$, with the aid of a rod linker in PBS assembly [7–10]. According to the spectrum properties of phycocyanins, they are classified into three types [2,7–10,13–14]: 1) C-phycocyanin (C-PC) which is mainly observed in cyanobacteria [1–3,7–10,13–16]; 2) R-phycocyanin (R-PC) which is mainly come from red algae [7–10,13–18]; 3) R-phycocyanin II (R-PC II) which is identified from marine cyanobacterium *Synechococcus* species [19]. Each (αβ) monomer of C-PCs contains three chromophores of phycocyanobilin (PCB),

one of which is carried by an α subunit and the other two are carried by a β subunit. For R-PCs, an α subunit contains one PCB but a β subunit carries one PCB and one phycoerythrobilin (PEB). Accordingly, C-PCs usually show a one-peak absorption spectrum where the peak is commonly within 610–620 nm, whereas R-PCs have a two-peak absorption spectrum in which the peak at about 550 nm comes from PEBs and that at about 615 nm from PCBs [2,7–10,13–18].

In previous reports [20–21], an R-PC was prepared from the marine red macroalga *P. urceolata*. The R-PC extracted in diluting phosphate buffer [20] or in distilled water [21] was first isolated by the hydroxyapatite chromatography which was developed by a stepwise elution with phosphate buffers. Then the R-PC fraction was further purified by the gel filtration on Bio-Gel P300, and the gel filtration was repeated until the absorbance ratio (*A*618/*A*280) of the obtained R-PC was higher than 3.5. The purified R-PC showed two absorption peaks at 550 nm and 618 nm, and exhibited a strong fluorescent emission at 637 nm and a small one at 566 nm [20–22]. The R-PC contained two subunits, 18.2 kDa α and 19.4 kDa β which were determined on a Biflex III matrix assisted laser desorption/ionization-time of flight mass spectrometry (MALDI-TOF-MS); and it was assumed to take the form of a trimer $(\alpha^{18.2}\beta^{19.4})_3$ [22]. The α subunit was proved to have 162 residues and it carried one PCB chromophore at 84Cys (α84); the β subunit was composed of 172 residues and carried one PCB at 84Cys (β84) and one PEB chromophore at 155Cys (β155) [23]. In crystal, the R-PC composed of three (αβ) monomers assembled around a three-dimensional axis to form a disc-shaped $(\alpha\beta)_3$ trimer which showed about 11 nm in diameter, about 3 nm in thickness and a central cavity about 3.5 nm in diameter [23].

The present work is to investigate the phycobiliprotein components of the intact phycobilisomes prepared from the marine red macroalga *P. urceolata* by ultracentrifugation on step sucrose gradients [6], and it focuses on the component of R-PC that is a key constitute to the assembly and light energy transfer of the phycobilisomes from red algae, which are understood much less than the phycobilisomes from cyanobacteria, by functioning as the connecter between two PBS subdomains. From the phycobilisomes disassociated in diluting buffer, we isolated and purified an R-PC component by a process in which two modes of chromatography were combined with native PAGE. In contrast with the previously reported R-PC from the phycobiliprotein extract [20–23], the R-PC prepared from the phycobilisomes was proved to have one α subunit and two β subunits that were different both in molecular mass and in pI. The heterogeneous composition of the β subunits reveals that the R-PC may have one more forms of trimeric complexes different in β subunit combinations. These researches are favorable to a deeper understating of R-PC complexes about their structure and function in the assembly of phycobilisomes from red macroalgae.

Materials and Methods

1. Preparation of the Phycobilisome

P. urceolata used as an organism material in this work for R-PC study is a widespread red macroalga and not a protected organism species. It grows luxuriantly on the local seaside around Yantai city in the area of Northern Yellow Sea of China. The alga specimens were collected at the beach within the district under the jurisdiction of the city government. For the marine algae which are not protected organism species and are used as samples only for teaching and academic or scientific investigations, there are no regulations on the specimen collection limitation of them from the city government and other organizations of biological diversity protection. Therefore, the specimen collection of *P. urceolata* used in the research is not required to apply for a specific permission to government departments or related organizations. In addition, there are no other protected organism species included in this investigation.

P. urceolata is abundant from February to April; during this time, temperature of the seawater where the alga grows rises from about 5 to 15°C [6]. After rinsed by seawater, the alga sample was suspended in 900 mM phosphate buffer (pH 7.0) of 300 ml per 100 g fresh alga, and then the sample was ultrasonicated by Ultrasonic Cell Disruptor (TY92-II, China) for ten min at room temperature. All phosphate buffers used in this work contain 4 mM sodium azide, 4 mM EDTA-Na unless they are specified. Phycobilisomes were solubilized off from thylakoid membranes with 2% NP-40 in 900 mM phosphate buffer by stirring gently for about 45–60 min at room temperature. After centrifugation of the PBS solution (CR22GII centrifuge, HITACHI) at 30,000 g for 20 min, the purple color supernatant in middle was collected as the PBS extract.

Intact phycobilisomes were purified from the PBS extract by ultracentrifugation on a step density gradient of sucrose [6]. The step sucrose gradients from low to high were 0.8 M, 1.0 M, 1.5 M and 2.0 M. The centrifugation was carried out at 132,000 g for 3.5 h at 20°C. The intact phycobilisomes in purple color were collected from the thick band located in the boundary between 1.0 M and 1.5 M sucrose layers.

2. Chromatography

The prepared phycobilisomes were dissociated in 50 mM phosphate buffer (pH 7.0) at 6°C for 12 h. Then R-phycocyanin was firstly isolated from the dissociated phycobilisomes by gel filtration on Sephadex G-150. The column (3.5 cm × 65 cm) of Sephadex G-150 was eluted with 50 mM phosphate buffer (pH 7.0) at a flow of 30 ml/h. The blue-violet fraction of R-PC was collected, and stored at 6°C in dark.

The collected R-PC was directly applied to an ion exchange column of DEAE-Sepharose Fast Flow (2.6 cm × 10 cm). The column was pre-equilibrated with 25 mM phosphate buffer (pH 7.0) and the sample was loaded on at a flow of 15 ml/h. The loaded column was washed with 25 mM phosphate buffer of about 10-fold column volume at 30 ml/h so that the proteins unbound were eluted out of the column. The chromatography on DEAE-Sepharose Fast Flow was developed by a linear ion strength gradient of NaCl from 50 mM to 400 mM in 25 mM phosphate buffer (pH 7.0) of 500 ml at an elution flow of 30 ml/h.

3. Gel Electrophoresis

Native-PAGE was composed of 6.5% (w/v) separating gel in pH 7.5 Tris-HCl buffer and 3% (w/v) stacking gel in pH 5.5 Tris-phosphate acid buffer, and Tris-Barbital of 0.01 M was used as electrode buffer (pH 7.0) [24]. The PAGE was performed with constant current of 2 mA per track when the samples run in the stacking gel, and after the samples entered the separation gel the current was adjusted up to 4 mA per track. After the electrophoresis, the slab gel was stained in Coomassie Blue G-250 solution for examining colorless polypeptides [6,24].

Sodium dodecyl sulfate (SDS)-PAGE was employed to analyze subunit components of the obtained R-PC (6,24). The polypeptide analysis of the obtained R-PC was fulfilled by a gradient SDS-PAGE which had a separating gel of 12–21% in pH 8.8 Tris-HCl buffer and a stacking gel of 4% in pH 6.8 Tris-HCl. Tris-Gly buffer (pH 8.3) of 0.1 M which contained 0.2% (w/v) SDS was employed as electrode buffer. The SDS-PAGE was carried out with a constant current of 1–2 mA per track for about 2–3 h. After

the electrophoresis, the slab gel was stained successively with $Zn(SO_4)_2$ and Coomassie Blue G-250.

Native isoelectric focusing (IEF) was performed in a range of pH from 4.0 to 6.5 on a polyacrylamide gel with T = 5.5% and C = 3%. Denature IEF was carried out in a pH range from 3 to 10 on a polyacrylamide gel with T = 7% and C = 3%. Riboflavin of 0.4 µg/ml was employed to catalyze polymerization of the polyacrylamide gels under cool light so as to overcome the fact that polyacrylamide gel is not easy to polymerize under acidic conditions. For the both IEFs, anode solution was 0.5 M phosphoric acid, whereas HEPES Free Acid of 0.4 M was used as cathode solution for the native IEF and ethanolamine of 0.5 M for the denaturing IEF.

Samples used for denaturing IEF were prepared as following steps: 1) samples were denatured and precipitated in 20% (w/v) trichloroacetic acid, and then collected by centrifugation at 20,000 g for 15 min; 2) the denatured proteins were washed with 4 mM EDTA-Na_2 containing 10% (v/v) glycerol for the remains of trichloroacetic acid to be removed, and the polypeptides were collected again by centrifugation at 20,000 g for 15 min after each washing; 3) finally, the polypeptides in denaturing situation were fully suspended in a sample preparation solution which was composed of 2% (v/v) Ampholine, 0.5% (v/v) NP-40 (or Triton X–100), 120 mM mercaptoethanol,8 M Urea and 0.003% (w/v) bromophenol blue. The suspended polypeptides were centrifuged at 20,000 g for 15 min so that insoluble substances were removed. The supernatant was used as the polypeptide samples prepared for denaturing IEF.

4. Spectrum Measurement

Absorption spectra of phycobilisome and phycobiliprotein samples from corresponding preparation steps were recorded in pH 7.0 phosphate buffer with UV-1900 spectrometer. Fluorescence spectra of the samples were examined with Cary Eclipse fluorescence spectrometer in pH 7.0 phosphate buffer.

5. Mass Spectrum Measurements

Subunits in gels from SDS-PAGE were digested with Trypsin (10 ng/µl) in 25 mM ammonium carbonate at 37°C overnight, and the peptides from the digested subunits were extracted in turn with 5% (v/v) trifluoroacetic acid and 50% (v/v) acetonitrile in 2.5% (v/v) trifluoroacetic acid. The peptides resuspended in 0.1% (v/v) trifluoroacetic acid was used as samples for mass spectrum measurements, and α-cyano-4-hydroxycinnamic acid (α-CHCA) of 7 mg/ml in 50% (v/v) acetonitrile and 0.1% (v/v) trifluoroacetic acid was employed as the matrix. The peptide fragments of the individual subunits were analyzed by MALDI-TOF mass spectrometry (UltrafleXtremeTM MALDI-TOF/TOF, Bruker Daltonics In.). The parameters set for the mass spectrum measurement were: laser light: 337 nm; acquisition mode: shots/sub-spectrum 50, total shots/spectrum 1000; resolution: 50,000; mass tolerance: ±0.5–0.1 Da; Min S/N: 100; mass range: from 500 Da to 3500 Da.

Results

1. The Preparation of Phycobilisomes

The phycobilisomes in purple color were layered in the sucrose gradients between 1.0 M and 1.5 M after the ultracentrifugation at 132,000 g for 3.5 h at 20°C (Figure 1a), and the layers above the PBSs in red and yellow-green color were dissociated phycobilisomes and chlorophyll-containing substances, respectively. Absorption spectrum of the prepared phycobilisomes (Figure 1b) showed that the PBSs had three absorption peeks at 500 nm, 540 nm and 567 nm, and two weak absorption shoulders at 616 nm and 650 nm. As showed in fluorescent emission spectrum of the PBSs (Figure 1b), there were two fluorescent emission maxima: the weak at 578 nm and the strong at 672 nm, revealing the directional energy transfer from rod R-PEs to core APs in high efficiency. These features demonstrated that the prepared phycobilisomes were intact.

2. The Preparation of R-phycocyanins

R-phycocyanins were firstly isolated by the gel filtration on Sephadex G-150 from the phycobilisomes dissociated by dialyzing to 50 mM phosphate buffer (pH 7.0) at 4°C overnight. As shown in Figure 2a, the chromatogram of the gel filtration developed with 50 mM phosphate buffer (pH 7.0) at a flow of 30 ml/h exhibited that the dissociated phycobilisomes were separated into four fractions: R-phycoerythrin, R-phycocyanin, some substances in light red color and some chlorophyll-containing complexes (Figure 2a). The absorption spectrum of the R-PC fraction was showed in Figure 2b. The absorption maximum of the spectrum at 620 nm originates from the phycocyanobilin of R-PC, the absorption peaks at 499, 546 and 565 nm indicate the existence of R-PEs, and the absorption shoulder at about 650 nm suggests that the R-PC fraction contains AP proteins.

The R-PC fraction collected from the Sephadex G-150 chromatography was directly loaded on the ion exchange column of DEAE-Sepharose FF, and the column was developed with the linear gradient of NaCl concentration in 25 mM phosphate buffer (pH 7.0). As shown in Figure 3a, the elution curve exhibited three peaks; the elution volume of R-PC fraction was from 260 ml to 320 ml, corresponding to NaCl gradient from 232 mM to 274 mM. Obviously, the R-PC was efficiently separated from R-PEs (Figure 3a) by the ion exchange chromatography.

The absorption and corresponding second derivative spectra of the R-PC fraction from the ion exchange chromatography were showed in Figure 3b and 3c. The absorption maximum at 617 nm is attributed to the phycocyanobilins carried by both α and β subunits and that at 549 nm to the phycoerythrobilins carried only by β subunits. The small absorption shoulder at about 500 nm (Figure 3b) and the fluorescent emission at 573 nm (Figure 3c) indicated the existence of R-PEs in the R-PC fraction.

Examined by the native-PAGE with 6.5% (w/v) separation gel in pH 7.5 Tris-HCl buffer, the R-PC fraction from the ion exchange chromatography showed a strong red fluorescent band under UV-light at 365 nm (Figure 4 lane 2 (left)), but it also showed a thin blue band after the gel was stained with Coomassie Blue G-250 (Figure 4 lane 2 (right)). The thin blue band was identical to that of purified R-PE (Figure 4 lane 1 and lane 2). This demonstrated that the prepared R-PC still had the trace of R-PE contamination, which was consistent with the conclusion from the spectral properties of the R-PC fraction (Figure 3b and 3c). Accordingly, the R-PC from the ion exchange chromatography was further purified by the native PAGE performed in the neutral buffer system and the R-PC obtained by the native-PAGE showed a single band at about pH 5.7 in the native IEF with a pH range from 4.0 to 6.5 (Figure 4 lane 3 and lane 4). Figure 5 showed the spectral properties of the PAGE-purified R-PC: there were two absorption peaks at 549 nm and 617 nm (Figure 5a), and one fluorescent emission peak occurred at 636 nm (Figure 5b); moreover, no R-PE absorption and fluorescence were observed again. Molecular mass measurement of the PAGE-purified R-PC by gel filtration on Superdex 200 (not shown) showed that the R-PC was 136 kDa, which indicated that it was a trimer.

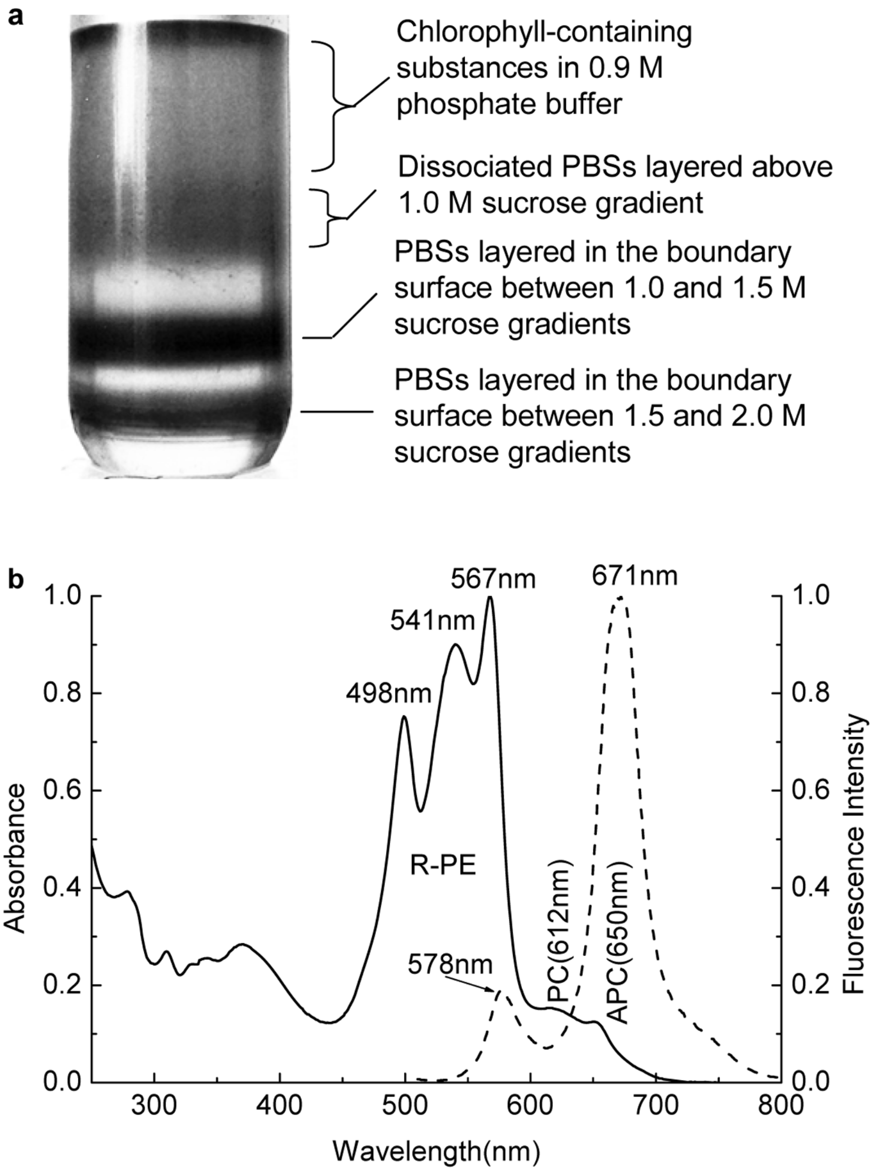

Figure 1. Preparation and spectral properties of the phycobilisomes from the red macroalga *Polysiphonia urceolata*. The phycobilisomes were prepared by sucrose step-gradient ultracentrifugation (a): a large fraction of phycobilisomes was situated within the boundary between 1.0 M and 1.5 M sucrose layers, and a small one was situated in the boundary between 1.5 M and 2.0 M sucrose layers; the former was used as samples for phycobiliprotein isolation and purification. Absorption (b solid line) and fluorescence emission (b dash dot line) spectra of the prepared intact phycobilisomes were recoded in pH 7.0 phosphate buffer at room temperature in which the phycobilisomes were excited at 498 nm.
doi:10.1371/journal.pone.0087833.g001

3. The Polypeptide Composition of the Prepared R-PC

Polypeptide components of the R-PC purified by the native-PAGE were analyzed by the SDS-PAGE with a gradient separation gel of 12–21% in pH 9.0 Tris-HCl buffer. As shown in Figure 6 (lane 1 and 2), after the gel was stained with $Zn(SO_4)_2$, on the gel slab there occurred four fluorescent bands of chromophore-charring subunits under UV-light at 365 nm(Figure 6 lane 2). The four fluorescent bands showed exactly equivalent to four blue bands after the gel was stained with Coomassie Blue G-250 and no other band without fluorescence was observed (Figure 6 lane 1). The four bands located at 17.5 kDa, 21.3 kDa, 22.6 kDa and 40.8 kDa, respectively. The three former bands corresponded with three subunits, $\alpha^{17.5}$ (α), $\beta^{21.3}$ (β) and $\beta^{22.6}$ (β'), and the two β subunits exhibited about equal content; whereas the last one was proved to be a subunit complex which was not completely dissociated. These results indicate that the purified R-PCs are composed of three chromophore-carrying subunits, $\alpha^{17.5}$, $\beta^{21.3}$ and $\beta^{22.6}$, and contain no colorless linker polypeptide. As a trimer, therefore, the R-PCs may exist in two trimeric aggregates, $(\alpha_3^{17.5}\beta_3^{21.3})$ and $(\alpha_3^{17.5}\beta_3^{22.6})$ or $(\alpha_3^{17.5}\beta_2^{21.3}\beta^{22.6})$ and $(\alpha_3^{17.5}\beta^{21.3}\beta_2^{22.6})$. However, the prepared R-PCs were showed only one band at about pH 5.7 in the native IEF with a pH range from 4.0 to 6.5 (Figure 4 lane 3 and 4) and not definitely resolved into one more bands. This reveals at least that the different forms of the trimeric R-PCs have their pIs much too close to be clearly separated by native IEF.

In the denaturing IEF with the gel concentration of 7% in a pH range from 3.0 to 10.0, as shown in Figure 6 lane 3 and lane 4, the

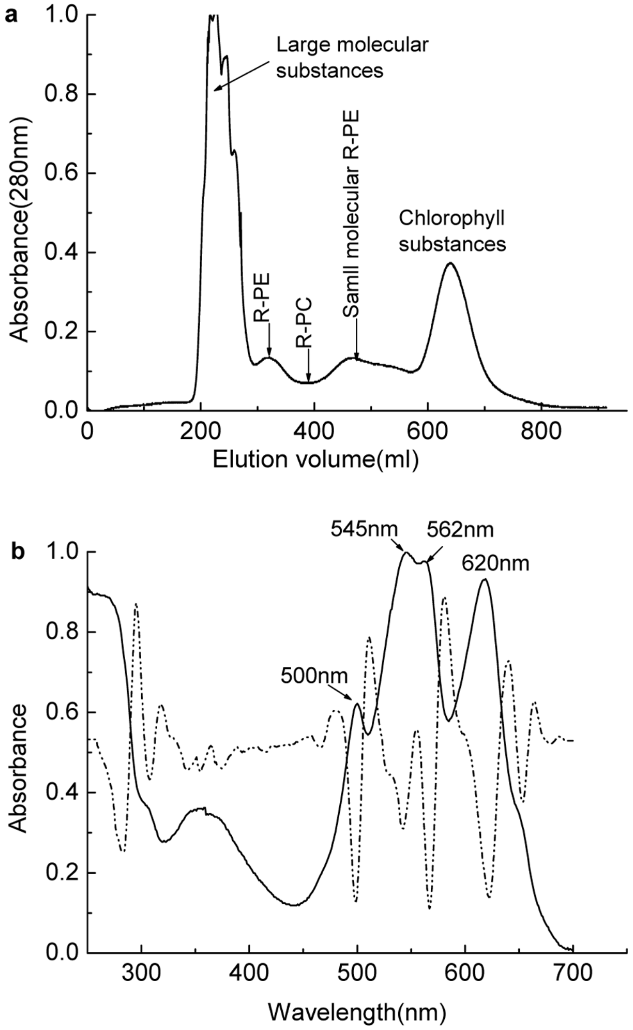

Figure 2. Isolation of the R-phycocyanin by gel filtration and spectral properties of the isolated R-phycocyanin. (a) the chromatogram of the R-phycocyanin isolation from the dissociated phycobilisomes by the **Sephadex G-150**gel filtration and (b) the absorption (solid line) and second derivative (dash dot line) spectra of the obtained R-phycocyanin fraction. The gel column (3.9 cm×65 cm) was eluted with 50 mM phosphate buffer (pH 7.0) at 30 ml/h.
doi:10.1371/journal.pone.0087833.g002

three subunits of the obtained R-PC exhibited fluorescent bands under UV-light at 365 nm (Figure 6 lane 3), and native color of the band at pH 6.4 was blue and that of the other two at pH 5.4 and 5.3 was purplish red. After the gel was stained with Coomassie Blue G-250 (Figure 6 lane 4), the three subunit bands in blue color were identical to those (Figure 6 lane 3) under the UV-light. Absorption spectra of the three subunits (Figure 7) demonstrated that the subunit with pI 6.4 showed a strong absorption band from chromophore PCB at about 597 nm and the other two subunits with pI 5.3 and 5.4 exhibited a strong absorption band from PEB at about 553 nm as well as an absorption shoulder from PCB at about 600 nm. As shown in Figure 8, the 2D-PAGE analysis of the R-PC subunits demonstrated that the subunit with pI 6.4 was $\alpha^{17.5}$, and the subunits with pI 5.4 and pI 5.3 were $\beta^{22.6}$ and $\beta^{21.3}$, respectively.

Fig. 9 gave the mass spectra of the two β subunits obtained by MALDI-TOF mass spectrometry. The mass spectra adequately exhibited the differences of $\beta^{22.6}$ (Fig. 9 a) and $\beta^{21.3}$ (Fig. 9 b) in *m/*

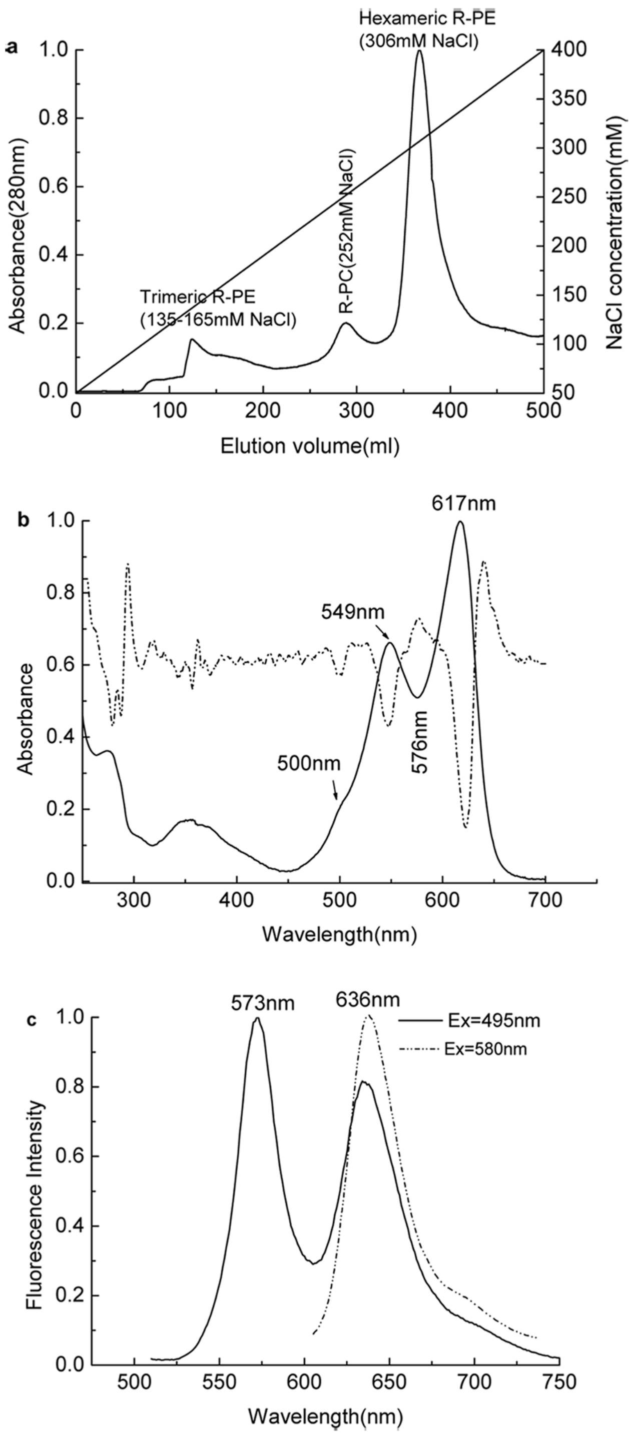

Figure 3. Purification of the R-phycocyanin by ion exchange chromatography and spectral characteristics of the purified R-phycocyanin. (a) The chromatogram of R-phycocyanin purification from the gel filtration fraction by ion exchange chromatography on DEAE-Sepharose Fast Flow, (b) absorption (solid line) and second derivative (dash dot line) spectra of the obtained R-phycocyanin fraction and (c) the fluorescence emission spectra of the R-phycocyanin fraction at room temperature when the fraction was excited at 495 nm (solid) and at 580 nm (dash dot). The ion exchange column (2.6 cm×10 cm) was eluted with the NaCl ion-strength gradient from 50 mM to 400 mM in 25 mM phosphate buffer (pH 7.0) at 30 ml/h.The spectra were recorded in pH 7.0 phosphate buffer.
doi:10.1371/journal.pone.0087833.g003

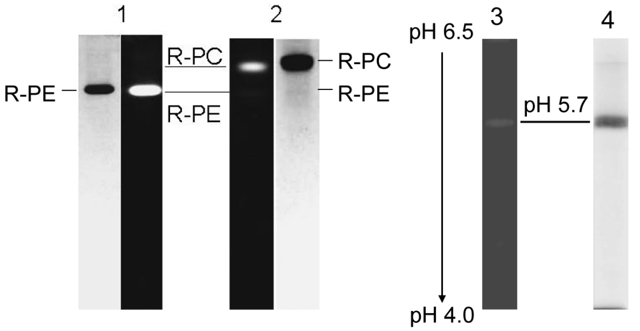

Figure 4. PAGE and IEF analysis of the R-phycocyanin purified by the chromatography on DEAE-Sepharose Fast Flow. The native-PAGE of the purified R-PE (lane 1) and the R-PC (lane 2) purified by the ion exchange chromatography and the native-IEF (lane 3 and 4) of the R-PC prepared by the native-PAGE: the PAGE with a separating gel of 6.5% was performed in a neutral pH system, and the gel was stained with Coomassie Blue G-250 after it was imaged under UV-light at 365 nm; the native-IEF with a gel of 5.5% was performed in a pH range from 4.0 to 6.5, and the gel was visualized under UV-light at 365 nm (lane 3) and by staining with Coomassie Blue G-250 (lane 4).
doi:10.1371/journal.pone.0087833.g004

z peaks. For example, within the range of m/z from 1000 to 1300, $\beta^{22.6}$ showed two m/z peaks respectively at 1127.467 and 1283.714 but $\beta^{21.3}$ had one at 1077.593; within the range from 1400 to 1600, two peaks occurred at 1449.747 and 1567.745 for $\beta^{22.6}$ with respect to one peak of $\beta^{21.3}$ at 1417.743; moreover, the peaks of $\beta^{22.6}$ and $\beta^{21.3}$ also exhibited the differences within the ranges from 1600 to 2000 and 2400 to 2800. These mass spectrum results further demonstrated that $\beta^{22.6}$ and $\beta^{21.3}$ were two different subunits of the prepared R-PC trimer.

The polypeptide analysis by the gradient SDS-PAGE determined that the PAGE-purified R-PC contained the one α subunit and the two β subunits (Figure 6). However, the difference of the two β subunits in apparent molecular mass (about 1.3 kD) may not make the prepared R-PCs show their trimeric aggregates different in molecular size to a degree large enough for them to be resolved into two groups by gel filtration on Superdex 200. The characterization of the R-PC subunits by the 2D-PAGE (Figure 8) demonstrated that the α subunit was homogeneous in apparent molecular mass ($\alpha^{17.5}$) as well as in isoelectric point (pI 6.4), whereas the two β subunits were different not only in apparent molecular mass ($\beta^{22.6}$, $\beta^{21.3}$) but also in isoelectric point (pI 5.4, pI 5.3). But the difference of the two β subunits in pI (about 0.1 pH) is seemingly not make the prepared R-PC trimers have their electronic charge variation large enough for them to be separated into one more bands by the native PAGE performed in neutral buffer system (Figure 4 lane 2) and have their pI differences large enough for them to be clearly resolved into one more groups by the native IEF (Figure 4 lane 3 and lane 4).

Discussion

R-PC in the phycobilisome from red algae, especially red macroalgae which grow in natural environments, is a minor biliprotein component relative to the major component of R-PE. By the methods of biliprotein estimation summarized by Rowan [25], based on the PBS absorption spectrum (Figure 1b) the three types of phycobiliproteins in the phycobilisomes of *P. urceolata* are estimated to have approximate proportions: hexameric R-PE: trimeric R-PC: trimeric AP = 7: 1: 2. The proportion of 7: 1 makes the R-PE hexamers be the key component to influencing

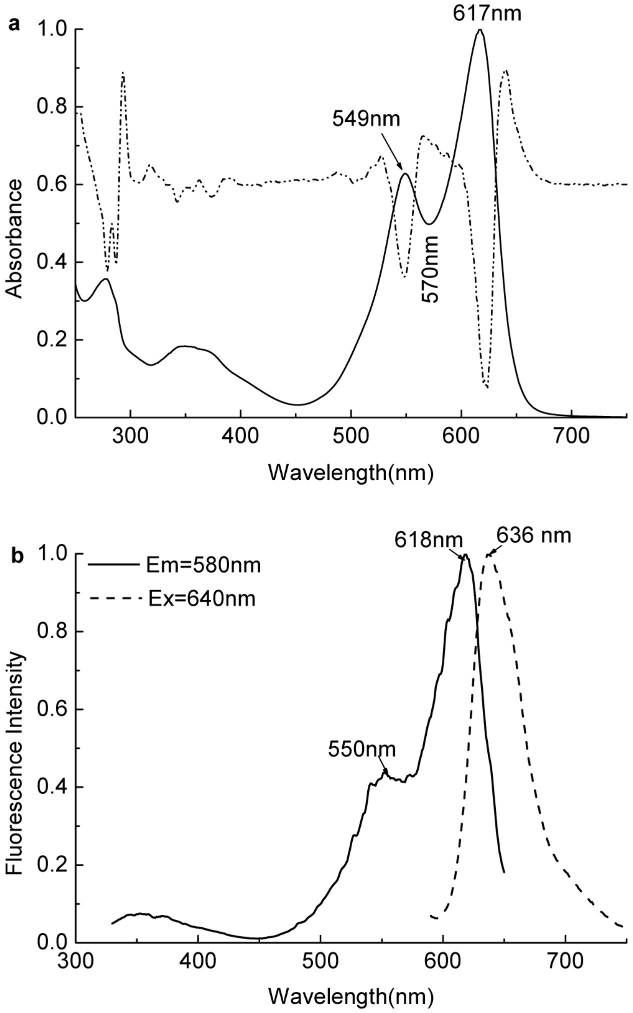

Figure 5. Spectral properties of the R-phycocyanin purified by the native-PAGE performed in a neutral pH system. The absorption (a solid line) and second derivative (a dash dot line) spectra of the purified R-PC in was recorded in pH 7.0 phosphate buffer. The fluorescence excitation (b solid line) and emission (b dash line) spectra of the R-PC were recorded at room temperature in pH 7.0 phosphate buffer respectively on the examination at 640 nm and the excitation at 580 nm.
doi:10.1371/journal.pone.0087833.g005

the trimeric R-PC preparation from *P. urceolata* phycobilisomes: they may cause large interference with the efficiency of R-PC preparation in chromatographic procedures, and in the meantime bring contaminants to the individual R-PC fractions obtained from each step of the preparation procedures. If the purpose to prepare R-PC from the phycobilisomes of red algae is to investigate the properties and functions of R-PCs on PBS assembly, it is necessary to have the prepared R-PC to the most degree maintain its natural status identical to that in phycobilisomes. For this reason, the preparation process, especially main isolation and purification procedures, needs to be designed and performed under conditions as mild as possible.

In the present work, the three main methods which were elaborately selected and designed were employed for the R-PC preparation from the intact phycobilisomes of the marine red macroalga *P. urceolata*: the gel filtration on Sephadex G-150 developed with pH 7.0 phosphate buffer of 50 mM, the ion exchange chromatography on DEAE-Sepharose FF developed by

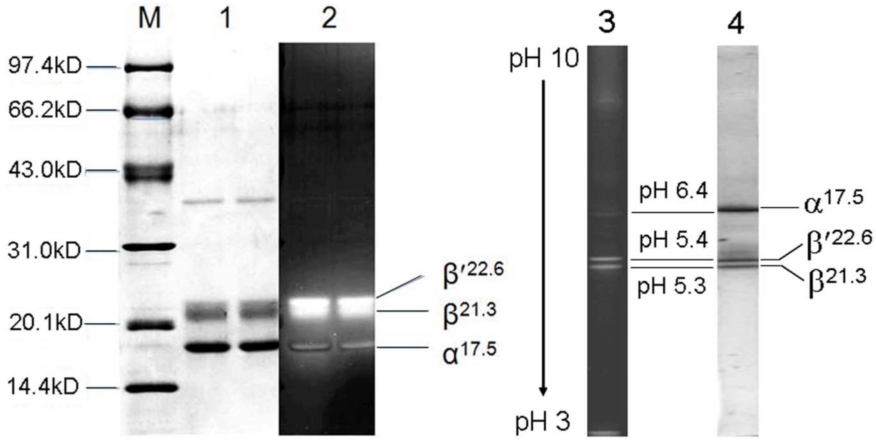

Figure 6. Polypeptide analysis of the prepared R-phycocyanin by SDS-PAGE and denaturing IEF. The SDS-PAGE (lane 1 and 2) had a gradient separating gel from 12% to 21% in pH 9.0 Tris-HCl buffer and the denaturing-IEF (lane 3 and 4) was a gel of 7% in a pH range from 3.0 to 10. The SDS-PAGE gel was examined under UV-light at 365 nm after it was stained with $Zn(SO_4)_2$ (lane 2) and then by staining with Coomassie Blue G-250 (lane 1). The denaturing-IEF gel was visualized under UV-light at 365 nm (lane 3) and by staining with Coomassie Blue G-250.
doi:10.1371/journal.pone.0087833.g006

a linear ionic strength gradient of NaCl in 25 mM phosphate buffer (pH 7.0) and the native PAGE performed in a neutral buffer system. Of the three procedures, the gel filtration is the mildest of all chromatography techniques because there is almost no interaction between gel particles and proteins, compared with hydroxyapatite chromatography the chromatography on DEAE-Sepharose FF is much milder because the ion exchanger with minimized non-specific interactions with sample components couples with acidic proteins like phycobiliproteins only simplest by static electronic charges, and the native PAGE performed in a neutral buffer system is more favorable to maintaining the stability of R-PC complexes by preventing them from situating in alkaline buffer systems because the R-PCs of *P. urceolata* disassociate easily under alkaline conditions. These procedures give adequate foundations for accomplishing the preparation of the P-PC

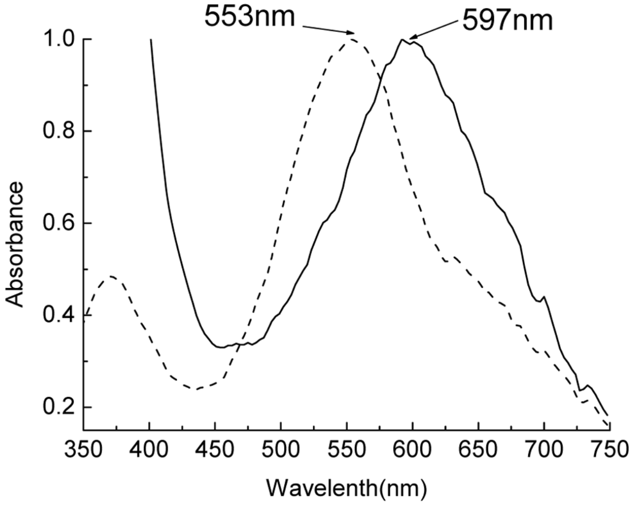

Figure 7. Absorption spectra of the R-phycocyanin subunits. The subunit with its pI of 6.4 (solid line) and the two subunits with their pIs of 5.4 and 5.4 (dash line) were obtained from the denaturing-IEF in a pH range from 3.0 to 10, and the spectra were measured by UV-microspectrometer (NanoDrop-1000).
doi:10.1371/journal.pone.0087833.g007

complexes which are distinct in subunit composition from the P-PCs previously reported from *P. urceolata*.

Unlike the R-PC preparation reported in the previous work [20–22], here the minor R-PCs in the dissociated phycobilisomes were first separated from most of the major R-PEs by the gel filtration on Sephadex G-150 based on the difference in molecular size between trimeric R-PC and hexameric R-PE. Owing to its low content in the dissociated phycobilisomes, the R-PC fraction hardly showed elution peak in the chromatogram of the gel filtration when the elution was monitored by protein absorption at 280 nm (Figure 2a), but the R-PC fraction could conveniently be resolved by its specific absorption at about 617 nm. Compared with the PBS absorption spectrum (Figure 1b), the spectrum of the isolated R-PC confirmed that most of the R-PE was removed by the gel filtration. However, the R-PC fraction not only inevitably still contained a small number of the R-PE hexamers, but also had some R-PE which has been proved to exist in the dissociated phycobilisomes in their aggregates smaller than trimers. The existence of R-PEs in forms smaller than hexamer is characteristic of *P. urceolata* phycobilisome.

The R-PC sample in 25 mM phosphate buffer diluted from 50 mM was directly loaded on the DEAE-Sepharose column equilibrated with 25 mM phosphate buffer (pH 7.0), and the column was eluted with a NaCl linear gradient of 0.7 mM/ml (or 0.35 mM/min). One R-PE fraction eluted before the R-PC at about 150 mM NaCl was proved to be the R-PEs smaller than trimers, and the other R-PE fraction eluted after the R-PC at about 306 mM NaCl was the R-PE hexamers rich in the phycobilisomes (Figure 3a). This process where the gel filtration was combined with the ion exchange chromatography accords with the principle of multidimensional chromatography [26] and is verified to be convenient and efficient for the R-PC preparation from the dissociated phycobilisomes of *P. urceolata*.

The R-PC fraction from the ion exchange chromatography still contained a trace number of R-PEs. This was evidently indicated by the characteristic fluorescent emission of R-PEs at 577 nm and by the very small absorption shoulder at about 500 nm (Figure 3b and 3c). Moreover, the R-PE contaminant also caused the absorption valley of R-PC to be red-shifted from 570 nm (Figure 3a) to 576 nm (Figure 5a). The existence of the thin R-PE band revealed by the native PAGE with a neutral buffer system

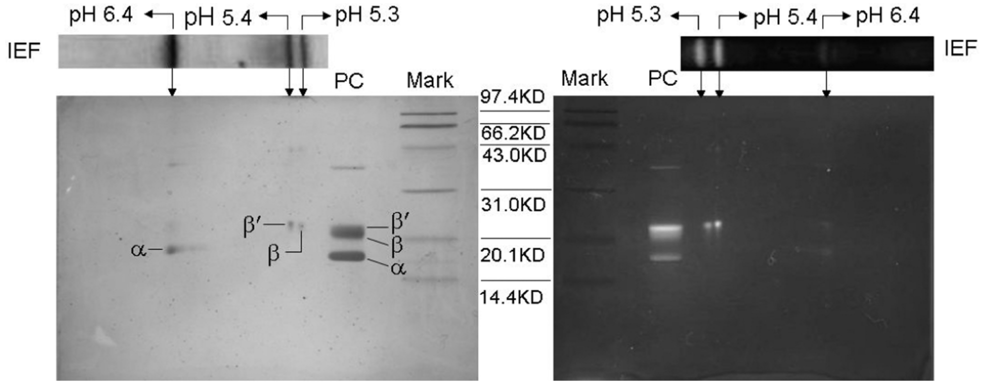

Figure 8. The SDS-PAGE of the R-PC subunits after the denaturing-IEF. The SDS-PAGE with a separating gel of 16% was carried out in pH 8.8 Tris-HCl buffer, and then the gel was imaged by staining with $Zn(SO_4)_2$ (right) and silver (left), respectively.
doi:10.1371/journal.pone.0087833.g008

(Figure 4 lane 2) definitely supported the conclusion derived from the spectral features of the R-PC fraction. In contrast, the R-PC purified by the native PAGE exhibited no fluorescent emission at 577 nm and absorption shoulder at about 500 nm (Figure 5), demonstrating that the R-PE contaminant was adequately eliminated. Based on the present work, it has to be accentuated that the R-PC from *P. urceolata* behaviors gradually fading during the native PAGE performed in alkaline buffer systems, indicating that the R-PC is easily denatured in alkaline environments. This was the reason for us to employ a native PAGE performed in neutral buffer systems.

In addition, Ma and coauthors reported that the R-PC prepared from *P. urceolata* and its β subunit showed a fluorescent emission band at 566 nm [22]. But this emission was not observed in the present work. There is an argument about the emission at 566 nm: the fluorescent band of the prepared R-PC trimer may originate from the coexistent β subunit which comes from partial dissociation of the R-PC trimers, especially in low concentration [10,12].

The SDS-PAGE with a gradient concentration of 12%–20% in pH 9.0 Tris-HCl buffer (Figure 6 lane 1 and lane 2) and the denaturing IEF in a pH range from 3.0 to 10 (Figure 6 lane 3 and lane 4) demonstrated that the R-PC purified by the native PAGE was composed of one α subunit of 17.5 kDa and two β subunits of 21.3 kDa and 22.6 kDa. The two β subunits, $\beta^{21.3}$ (β) and $\beta^{22.6}$ (β′), was approximately in an equal proportion considering that the R-PC subunits may have different stability in alkaline environments. The 2D-PAGE (Figure 8) demonstrated that $\alpha^{17.5}$, $\beta^{21.3}$ and $\beta^{22.6}$ showed pI 6.4, 5.3 and 5.4, respectively. Furthermore, the absorption spectra of the subunit bands from the denaturing IEF (Figure 6 lane 3 and lane 4) demonstrated that the two subunits with pI 5.3 and 5.4 carried both chromophore PCB and PEB, whereas the subunit with pI 6.4 merely contained PCB, supporting the 2D-PAGE. The fact that the R-PC prepared from the intact phycobilisomes of *P. urceolata* contains two different β subunits is a noticeable difference from the previously reported R-PC which was prepared from the phycobiliprotein extract of *P. urceolata* [20–23] as well as the R-PCs reported from other red algae [7,10,13–18]. The heterogeneous subunit composition of phycobiliproteins was reported in some previous papers on the subunit components of allophycocyanins from cyanobacteria, such as *Synechococcus* 6301 [27–28], *Synechocystis* 6701 [3,7,8,29–31], *Mastigocladus laminosus* [7,32–37], *Anabaena sp.* PCC 7120 [7,38,39] and *Myxosarcina concinna* [24]. However, there are few reports on heterogeneous subunit composition of phycocyanins and phycoerythrins.

Except that trimeric complexes of the purified R-PCs composed of $\alpha^{17.5}$, $\beta^{21.3}$ and $\beta^{22.6}$ exhibited single band in the native PAGE, they were also not definitely resolved into one more bands by the native IEF a (Figure lane 3 and 4 lane). This adequately demonstrated that although $\beta^{21.3}$ and $\beta^{22.6}$ showed differences in molecular mass and pI, the prepared R-PCs exhibited a nearly equal ratio of charge to mass in native PAGE and very close pIs in native IEF as well as a homogeneous molecular size in gel filtrations and very similar net charge features in ion exchange chromatography. These facts also revealed that the differences of the two β subunits in pI and molecular mass could hardly make trimeric aggregates of the purified R-PCs, in which the two β subunits exhibited equal ratios to α one, show distinctive enough for the trimeric R-PCs to be resolved into one more groups even by native IEF.

Between the two most possible trimeric pairs of the prepared R-PC complexes, $(\alpha_3^{17.5}\beta_3^{21.3})$ and $(\alpha_3^{17.5}\beta_3^{22.6})$, and $(\alpha_3^{17.5}\beta_2^{21.3}\beta_1^{22.6})$ and $(\alpha_3^{17.5}\beta_1^{21.3}\beta_2^{22.6})$, each pair of which exists in disk-shaped trimer forms with equal ratios of individual β to α and shows positively no difference in molecular size in gel filtrations, and each of which is composed of $\alpha^{17.5}$ and one of the two β subunits ($\beta^{21.3}$ or $\beta^{22.6}$) more probably shows differences in pI between the two trimers to a certain extent enough for them to be separated by native IEF and even by native PAGE owing to the difference in pI between three $\beta^{21.3}$ and three $\beta^{22.6}$. In contrast, $(\alpha_3^{17.5}\beta_2^{21.3}\beta_1^{22.6})$ and $(\alpha_3^{17.5}\beta_1^{21.3}\beta_2^{22.6})$ may show differences in pI between $(\beta_2^{21.3}\beta_1^{22.6})$and $(\beta_1^{21.3}\beta_2^{22.6})$ so little that they could not be resolved into two distinct groups even by the native IEF in the pH range from 4.0 to 6.5 (Figure 4). Therefore, $(\alpha_3^{17.5}\beta_2^{21.3}\beta_1^{22.6})$ and $(\alpha_3^{17.5}\beta_1^{21.3}\beta_2^{22.6})$ are postulated to be the two forms of the R-PC trimers purified from the intact phycobilisomes of *P. urceolata*.

The feature that the proportion of R-PE hexamers to R-PC trimers is estimated to be 7:1 according to the absorption spectrum of the *P. urceolata* phycobilisomes (Figure 1b) suggests that each rod domain of the prepared phycobilisomes may consist of one copy of R-PC trimer and seven copies of R-PE hexamers. The R-PC trimer attaches on the AP core of phycobilisomes with the aid of a rod-core linker polypeptide, one R-PE hexamer stacks on the R-PC trimer by face to face with the aid of a PE-PC rod linker, and the other six R-PE hexamers pile up in turn face to face with the aid of γ subunits or other rod linkers [10]. This differs from the common understanding that phycocyanins take part in the

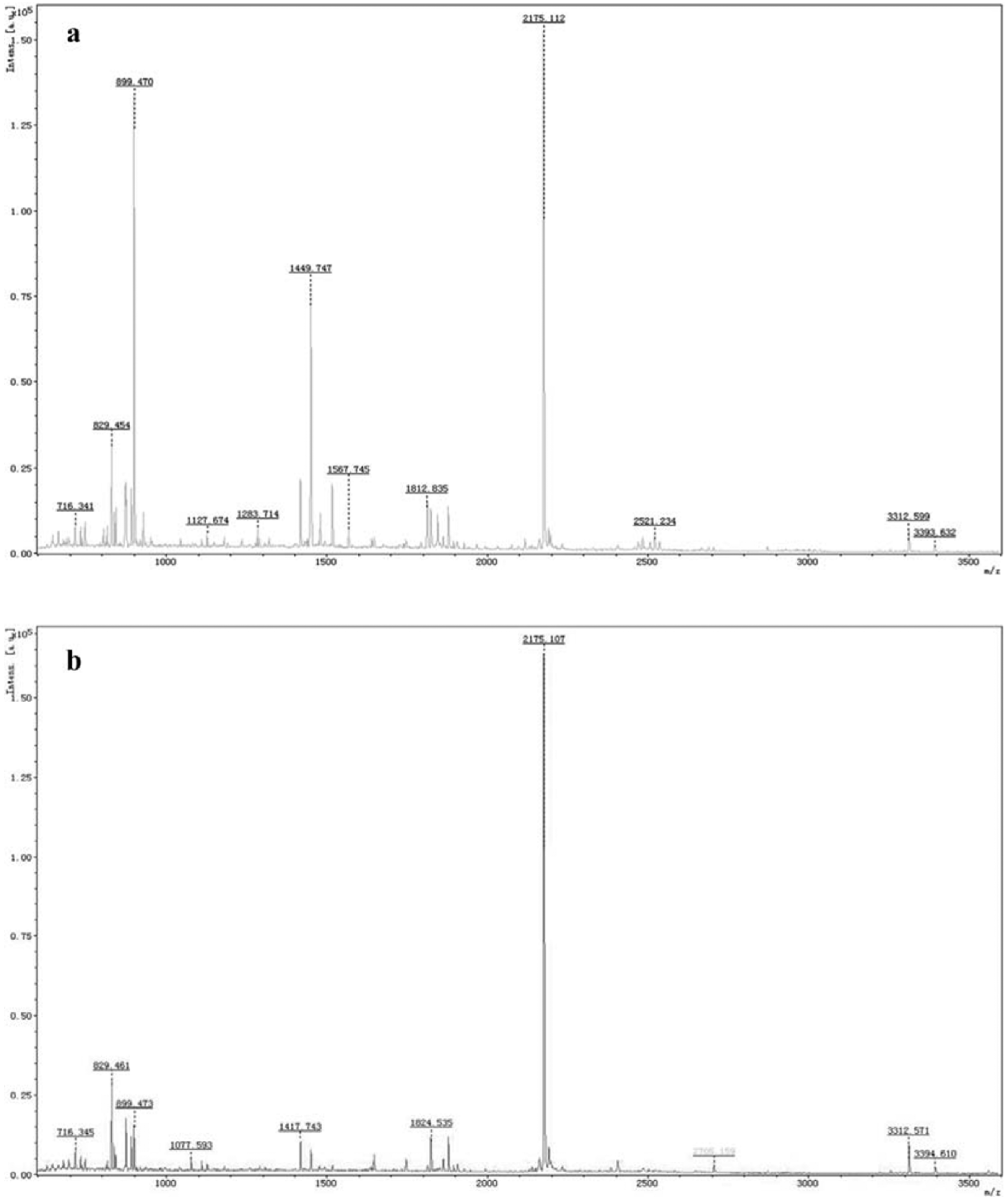

Figure 9. Mass spectra of the two β subunits. The two β subunits, $\beta^{22.6}$ (a) and $\beta^{21.3}$ (b), from the 2D-PAGE were digested with Trypsin in gel and the peptides ware analyzed by MALDI-TOF/TOF mass spectrometry in a mass range from 500 Da to 3500 Da.
doi:10.1371/journal.pone.0087833.g009

assembly of PBS rod domains in hexamer form [1,3,7–10]. Considering that an R-PC trimer is about 3 nm in thickness [23] and an R-PE hexamer is about 6 nm in thickness [40], the rod domain assembled by one copy of R-PC trimer and seven copies of R-PE hexamers may be about 45 nm in length. This length of the PBS rods is at least one time more than those reported from

cyanobacteria and red algae [41]. The high proportion of R-PE hexamers to R-PC trimers and the longer rod domains demonstrate that *P. urceolata* needs adequate R-PEs to harvest enough light for its optimal growth during its growing season from February to April, for R-PEs can more efficiently absorb the light from 450 nm to 570 nm in which chlorophylls poorly absorb and both of R-PCs and APs also less efficiently harvest. In this way, *P. urceolata* acclimatizes itself to the most favorable extent to the sun light of its living environments.

In conclusion, by contrast to the previous reports on the R-phycocyanins from *P. urceolata* [20–23] and other red algae [7,10,13,15–18], the results from this work have a few striking aspects which need to be accentuated: 1) the successful preparation of the R-PC with two different β subunits from the intact phycobilisomes of *P. urceolata* was accomplished by employing a process where the two chromatographic modes of gel filtration on Sephadex G-150 and ion exchange chromatography on DEAE-Sepharose FF were combined with the native PAGE performed in neutral buffer systems; 2) the prepared R-PC was determined to be composed of one α subunit ($\alpha^{17.5}$) and the two β subunits different in apparent molecular mass ($\beta^{21.3}$ and $\beta^{22.6}$), differing from the R-PC prepared from the phycobiliprotein extract of *P. urceolata* [20–23]; 3) the denaturing IEF and the two-dimension PAGE demonstrated that $\alpha^{17.5}$ has pI 6.4 and that the two β subunits of $\beta^{21.3}$ and $\beta^{22.6}$ with equal proportions have pI 5.3 and 5.4, respectively. This heterogeneous subunit composition has not been reported in previous papers on phycocyanins from red algae; 4) in the native IEF, the purified R-PC showed one band at pH about 5.7, which demonstrated that the R-PCs existed in the trimers similar to each other to such a degree that they behaved as the aggregates consistent in pI other than in the ratio of charge to mass and in molecule size; 5) on the basis of the present work, the two trimers are postulated to be ($\alpha_3^{17.5}\beta_2^{21.3}\beta_1^{22.6}$) and ($\alpha_3^{17.5}\beta_1^{21.3}\beta_2^{22.6}$) which are the most possible complexes to behave as the trimers of the prepared R-PCs homogeneous in pI other than in the ratio of charge to mass. These results can proffer a favorable reference for the preparation and characterization of R-PC from other marine red macroalgae, and furthermore they may also provide some certain advantages to the promotion of investigations on R-PC complexes and phycobilisome organization of red macroalgae.

Acknowledgments

We thank Prof. Min Chen for supporting this work, Wenjun Li for assistance with mass spectrometric measurement and Yang Wang for help with ultracentrifugation experiments.

Author Contributions

Conceived and designed the experiments: LS XF MZ. Performed the experiments: LW YQ. Analyzed the data: XF MZ SW LS. Contributed reagents/materials/analysis tools: XF MZ SW LS. Wrote the paper: LW LS.

References

1. Glazer AN (1984) Phycobilisome: a macromolecular complex optimized for light energy transfer. Biochim. Biophys. Acta 768: 29–51.
2. Glazer AN (1985) Light harvesting by phycobilisome. Annu. Rev. Biophys. Chem. 14: 47–77.
3. Glazer AN (1989) Light guides: Directional energy transfer in a photosynthetic antenna. J. Biol. Chem. 264: 1–4.
4. Galland-Irmouli AV, Pons L, Lucon M, Villaume C, Mrabet NT et al. (2000) One-step purification of R-phycoerythrin from the red macroalga *Palmaria palmate* using preparative polyacrylamide gel. J. Chromatography B 739: 117–123.
5. Holzwarth AR (1991) Structure-function relationships and energy transfer in phycobiliprotein antennae. Physiol. Plant 83: 518–528.
6. Sun L, Wang S, Gong X, Chen L (2004) A rod-linker-contained R-phycoerythrin complex from the intact phycobilisome of the marine red alga *Polysiphonia urceolate*. J. Photochem. and Photobiol. B: Biol. 76: 1–11.
7. Sidler WA (1994) Phycobilisome and phycobiliprotein structures. In: Bryant DA, editors. The molecular biology of cyanobacteria. Netherlands: Kluwer Academic Publication. 139–216.
8. MacColl R (1998) Cyanobacterial phycobilisomes. J. Struct. Biol. 124: 311–334.
9. de Marsac NT (2003) Phycobiliproteins and phycobilisomes: the early observations. Photosynthesis Research 76: 197–205.
10. Sun L, Wang S, Zhao M, Fu X, Gong X et al. (2009) Phycobilisomes from cyanobacteria. In: Gault PM, Maler HJ, editors. Handbook on Cyanobacteria: Biochemistry, Biotechnology and Application. New York: Nova Science Publishers. 105–160.
11. Kronick MN (1986) The use of phycobiliproteins as fluorescent labels in immunoassay. J. Immunol. Meth. 92: 1–13.
12. Glazer AN (1994) Phycobiliproteins-a family of valuable, widely use fluorophores. J. App. Phycology 6: 105–112.
13. Sun L, Wang S, Chen L, Gong X (2003) Promising fluorescent probes from phycobiliproteins. IEEE J. Selected Topics in Quantum Electronics 9: 177–188.
14. Glazer AN (1988) Phycobiliproteins. Methods in Enzymology 167: 291–303.
15. Bryant DA, Hixson CS, Glazer AN (1978) Structural Studies on Phycobiliproteins: III. Comparison of bilin-containing peptides from the β subunits of C-phycocyanin, R-phycocyanin, and phycoerythrocyanin. J. Biol. Chem. 253: 220–225.
16. Klotz AV, Glazer AN (1986) Phycobiliprotein-bilin linkage diversity: II. Structural studies on A- and D-ring-linked phycoerythrobilins. J. Biol. Chem. 261: 6797–6805.
17. Glazer AN, Hixson CS (1975) Characterization of R-phycocyanin: Chromophore content of R-phycocyanin and C-phycoerythrin. J. Biol. Chem. 250: 5487–5495.
18. Kursar TA, Meer JVD, Alberte RS (1983) Light-Harvesting System of the Red Alga *Gracilaria tikvahiael*: I. Biochemical analyses of pigment mutations. Plant Physiol. 73: 353–360.
19. Ong LJ, Glazer AN (1987) R-Phycocyanin II, a new phycocyanin occurring in marine *Synechococcus* species: Identification of the terminal energy acceptor bilin in phycocyanins. J. Biol. Chem. 262: 6323–6327.
20. Zeng F, Yang Z, Jiang L (1984) Isolation and characterization of R-phycocyanin from *Polysiphonia urceolata*, J. Hydrobiologia 116/117: 594–596.
21. Zhang JP, Wan ZL, Wang SG, Chang WR, Liang DC (1995) Isolation, crystallization and preliminary crystallographic analysis of R-phycocyanin from *Polysiphonia urceolata*. Acta Biophys. Sinica 11: 481–484.
22. Ma Y, Xie J, Zhang R, Hu C, Zhao J (2008) Molecular properties of R-phycocyanin subunits from *Polysiphonia urceolata* in potassium phosphate buffer. J Photochemical and Photobiological Sciences 7: 263–268.
23. Jiang T, Zhang J, Chang W, Liang D (2001) Crystal structure of R-phycocyanin and possible energy transfer pathways in the phycobilisome. Biophysical J. 81: 1171–1179.
24. Sun L, Wang S (2003) Allophycocyanin complexes from the phycobilisome of a thermophilic blue-green alga *Myxosarcina concinna* Printz. J. Photochem. Photobiol.: B Biol. 72: 45–53.
25. Rowan KS (1989) Photosynthetic pigments of algae. New York: Cambridge University Press.
26. Simpson RJ (2004) Purifying proteins for proteomics: A laboratory manual. Cold Spring Harbor: Cold Spring Harbor Laboratory Press.
27. Lundell DJ, Glazer AN (1983) Molecular architecture of a light-harvesting antenna: structure of the 18 S core-rod subassembly of the *Synechococcus* 6301 phycobilisomes. J. Biol. Chem. 258: 894–901.
28. Lundell DJ, Glazer AN (1983) Molecular architecture of a light-harvesting antenna: core substructure in *Synechococcus* 6301 phycobilisomes. J. Biol. Chem. 258: 902–908.
29. Glazer AN, Chan C, Williams RC, Sheila W, Clark JH (1985) Kinetics of energy flow in the phycobilisome core. Science 230: 1051–1053.
30. Glazer AN, Sheila W, Webb SP, Clark JH (1985) Disk-to-disk transfer as the rate-limiting step for energy flow in phycobilisomes. Science 227: 419–423.
31. Glazer AN, Clark JH (1986) Phycobilisomes: macromolecular structure and energy flow dynamics. Biophys. J. 49: 115–126.
32. Reuter W, Wehrmeyer W (1988) Core substructure in Mastigocladus laminosus phycobilisomes. Arch. Microbiol. 150: 534–540.
33. Reuter W, Wehrmeyer W (1990) Core substructure in phycobilisomes, II. The central part of the tricylindrical core-AP_{CM}-contains the "anchor" polypeptide and no allophycocyanin B. Arch. Microbiol. 153: 111–117.
34. Reuter W, Nickel-Reuter C, Wehrmeyer W (1990) Isolation of allophycocyanin B from *Rhodella violacea* results in a model of the core from hemidiscoidal phycobilisomes of rhodophyceae. FEBS Letters 273: 155–158.
35. Reuter W, Muller C (1993) Adaptation of the photosynthetic apparatus of cyanobacteria to light and CO_2. J. Photochem. Photobiol. B: Biol. 21: 3–27.
36. Reuter W, Nickel-Reuter C (1993) Molecular assembly of the phycobilisomes from the cyanobacterium *Mastigocladus laminosus*. J. Photochem. Photobiol. B: Biol. 18: 51–66.

37. Reuter W, Wiegand G, Huber R, Than ME (1999) Structural analysis at 2.2 Å of orthorhombic crystals presents the asymmetry of the allophycocyanin-linker complex, AP-L, from phycobilisomes of *Mastigocladus laminosus*. Proc. Natl. Acad. Sci. USA 96: 1363–1368.
38. Ducret A, Sidler W, Wehrli E, Frank G, Zuber H (1996) Isolation, characterization and electron microscopy analysis of a hemidiscoidal phycobilisome type from the cyanobacterium *Anabaena sp.* PCC 7120. Eur. J. Biochem. 236: 1010–1024.
39. Ducret A, Muller SA, Goldie KN, Hefti A, Sidler W et al. (1998) Reconstitution, characterization and mass analysis of the pentacylindrical allophycocyanin core complex from the cyanobacterium *Anabaena sp.* PCC 7120. J. Mol. Biol. 278: 369–388.
40. Chang W, Jiang T, Wan Z, Zhang J, Yang Z et al. (1996) Crystal structure of R-phycoerythrin from *Polysiphonia urceolata* at 2.8 Å resolution. J. Mol. Biol. 262: 721–731.
41. Su Hai-N, Xie Bin-B, Zhang Xi-Y, Zhou Bai-C, Zhang Yu-Z (2010) The Supramolecular Architecture, Function, and Regulation of Thylakoid Membranes in Red Algae: an Overview. Photosynth. Res. 106: 73–87.
